# Biocompatibility and Osteogenic Potential of Calcium Silicate-Based Cement Combined with Enamel Matrix Derivative: Effects on Human Bone Marrow-Derived Stem Cells

**DOI:** 10.3390/ma14247750

**Published:** 2021-12-15

**Authors:** Hye-Min Kim, Donghee Lee, Sin-Young Kim

**Affiliations:** 1 Department of Conservative Dentistry, Seoul St. Mary’s Hospital, College of Medicine, The Catholic University of Korea, 222 Banpo-daero, Seoul 06591, Korea; hmtoto@naver.com; 2 College of Medicine, The Catholic University of Korea, Seoul 06591, Korea; dong524@naver.com

**Keywords:** calcium silicate-based cement, Emdogain, mineral trioxide aggregate, human bone marrow-derived mesenchymal stem cell, biocompatibility, osteogenic potential

## Abstract

The characteristics of retrograde filling material are important factors that can affect the long-term success of apical microsurgery. Various calcium silicate-based cements (CSC) were introduced to overcome drawbacks of mineral trioxide aggregate (MTA), while Emdogain is known to be effective in the regeneration of periodontal tissues. The aim of this study is to evaluate the biocompatibility and osteogenic potential of various CSCs combined with Emdogain on human bone marrow-derived mesenchymal stem cells. Experimental groups were classified into eight groups depending on the material and the presence of Emdogain. In the cell-counting kit test, all experimental groups combined with Emdogain showed higher cell viability compared with those without Emdogain at days 1 and 2. In the wound-healing assay, cell migration increased significantly over time, with or without Emdogain. In the alkaline phosphatase assay, all groups treated with Emdogain showed higher activity compared with those without Emdogain at day 3 (*p* < 0.05). Using alizarin red S staining, all groups treated with Emdogain showed greater calcium nodule formation compared with those without Emdogain at days 7 and 14 (*p* < 0.05). In conclusion, using CSCs as retrograde filling materials and the application of additional Emdogain will increase bone regeneration and improve the prognosis of apical microsurgery.

## 1. Introduction

Tight apical sealing affects the success of apical microsurgery, and the regeneration of peripheral bone destroyed by apical inflammation is an important indicator of success [1,2,3]. Mineral trioxide aggregate (MTA) has excellent features, such as biocompatibility, odontoblastic ability, and tight sealing properties [4]. One of the representative retrograde filling materials, ProRoot MTA (Dentsply Tulsa Dental Specialties, Tulsa, OK, USA), induces the migration and proliferation of human bone marrow-derived mesenchymal stem cells (hBMSCs) [5]. Although it is known to be effective in the regeneration of periapical destructed bone, it has the disadvantages of discoloration, heavy metal content, and a long setting time [6,7,8,9,10]. Recently, various types of MTA and calcium silicate-based cements (CSCs) have been introduced and commercialized for effective sealing of root ends during apical microsurgery [4,11,12,13,14].

CSCs have been developed with the advantage of reduced setting time and no discoloration. They can be hardened in bleeding conditions and therefore applied more easily during retrograde filling procedures in apical microsurgery [15,16,17,18]. In particular, RetroMTA (BioMTA, Seoul, Korea) has been reported to be biocompatible and have adequate physical properties in comparison with ProRootMTA [19,20]. Endocem MTA Premixed (Maruchi, Wonju, Korea) is a recently developed material with a pre-mixed CSC included in a syringe, making it easier for the practitioner to apply it [21]. Enamel matrix derivative (EMD) is mostly composed of amelogenin and is known to contain growth factors. It has demonstrated significant potential in the regeneration of periodontal defects when applied to the root surface during periodontal surgery [22,23,24,25]. There are many recent reports regarding the use of Emdogain Gel (Straumann, Basel, Switzerland) in endodontics [26,27,28,29,30].

The aim of this study is to compare the biocompatibility and osteogenic potential of ProRoot MTA, RetroMTA, and Endocem MTA Premixed combined with Emdogain Gel on human bone marrow-derived stem cells (hBMSCs).

## 2. Materials and Methods

### 2.1. Human Bone Marrow-Derived Stem Cells (hBMSCs)

This study was approved by the Institutional Review Board of Seoul St. Mary’s Hospital, the Catholic University of Korea (IRB No. MC20SESI0067). Human mesenchymal stem cells derived from bone marrow (Catholic MASTER Cells) were procured from the Catholic Institute of Cell Therapy (CIC, Seoul, Korea). The cell line was anonymously established at the CIC, and second-generation Catholic Master Cells were used in this study. The cells were cultured in growth medium, which was made up of Hyclone 10% bovine serum (GE Healthcare Life Sciences), 100 U/mL of penicillin, and 100 μg/mL of streptomycin (α-MEM; GE Healthcare Life Sciences, Pittsburgh, PA, USA). Cell cultures were maintained in a humid environment at 37 °C with 5% CO_2_. In a colony-forming test, most hBMSCs maintained a fusiform morphology, which is constant with other kinds of mesenchymal stem cells. All experiments were accomplished under sterile state.

### 2.2. Production of Retrograde Filling Materials Disks

The retrograde filling materials evaluated in this study were ProRoot MTA, RetroMTA, Endocem MTA Premixed, and Emdogain Gel. Their composition is listed in Table 1. Each cement was mixed according to its manufacturer’s instructions. We made disk-shaped specimens with a diameter of 6 mm and a height of 3 mm for each cement, using a sterile rubber mold. All discs were sterilized for 4 h using ultraviolet rays on a clean bench after setting, covered with wet gauze at room temperature for 72 h.

### 2.3. Classification of the Groups

The experimental groups in this study were constituted as follows:

Group 1 (ProRoot MTA): The ProRoot MTA disks were made using the method described above. Growth medium (GM) was used to monitor cell viability and cell migration, and osteogenic medium (OM) was used in alkaline phosphatase (ALP) analysis and alizarin red S (ARS) staining assay to observe cell differentiation.

Group 2 (ProRoot MTA combined with Emdogain Gel): Emdogain was added to the GM or OM at a concentration of 100 μg/mL, and the experimental process was the same as for group 1.

Group 3 (RetroMTA): the experiment was conducted using RetroMTA disks in the same way as for group 1.

Group 4 (RetroMTA combined with Emdogain Gel): Emdogain was added to the medium at a concentration of 100 μg/mL, and the experimental process was the same as for group 3.

Group 5 (Endocem MTA Premixed): the experiment was conducted using Endocem MTA Premixed disks in the same way as for group 1.

Group 6 (Endocem MTA Premixed combined with Emdogain Gel): Emdogain was added to the medium at a concentration of 100 μg/mL, and the experimental process was the same as that used for group 5.

Group 7 (Control): the control group included hDPSCs cultured without experimental disks.

Group 8 (Emdogain Gel): Emdogain was added to the medium at a concentration of 100 μg/mL, and the experimental process was the same as that used for group 7. The Emdogain group included hDPSCs cultured with the above medium without experimental disks.

### 2.4. Cell Viability Measurement

A Cell Counting Kit test (CCK-8) (CK04-13; Dojindo, Kumamoto, Japan) was used to evaluate the cytotoxic effects of the three kinds of experimental disks. The cell proliferation rate of hBMSCs was analyzed shortly after incubation, and analyzed again 1, 2, and 4 days after incubation. hBMSCs were cultured at a density of 1.0 × 10⁴ cells/well in 24-well cell culture plates (SPL Life Sciences, Pocheon, Korea) in growth medium. After 24 h of incubation of the adherent cells, the optical density value was obtained.

Individual disks were placed on an insert (SPLInsert; SPL Life Sciences) with pores 0.4 mm in size, while the inserts were located above the hBMSCs. Each well was supplemented with 1 mL of additional medium in order to cover the top of the disk. hBMSCs cultured without experimental disks were used as a control group. Emdogain at the initial concentration of 30 mg/mL was added to groups 2, 4, 6, and 8, and diluted in α-MEM for experimental use at a final concentration of 100 μg/mL. A total of 20 μL CCK-8 solution was added to each well, and the plates were placed in an incubator at 37 °C for 1 h. Later, we rinsed each well with PBS, and added dimethyl sulfoxide for dissolving the synthesized formazam. Absorption at 450 nm was measured using an absorbance microplate reader (Power Wave XS; BioTek Instruments, Winoski, VT, USA). Each group was measured in octuplicate.

### 2.5. Cell Migration Assay

A wound-healing assay was used to evaluate cell migration ability. The hBMSCs were sprayed at a density of 3.5 × 10⁴ cells/well in growth medium in a 24-well plate. After 24 h of incubation, a 1000-μL pipette tip was used to create scratches in the middle of each adherent cell layer. After scratching, the detached cell debris was washed away with PBS. After an additional 24 h of incubation, each individual disk was placed on an insert in a well. Emdogain at the initial concentration of 30 mg/mL was added to groups 2, 4, 6, and 8, diluted in α-MEM to a final concentration of 100 μg/mL. hBMSCs were incubated for 4 days with the experimental disks, with the medium replaced every 48 h. Images of cell migration were collected at 0, 1, 2, and 4 days using a phase-contrast microscope (Olympus, Tokyo, Japan). We used ImageJ 1.46r software (National Institutes of Health, Bethesda, MD, USA) for measuring the surface area covered by cells. The degree of cell migration from both sides to the scratched region was calculated based on the area of initial scratched region. Each group was measured in quadruplicate.

### 2.6. Alkaline Phosphatase (ALP) Activity

The osteogenic potential of hBMSCs was evaluated on days 3 and 6, using an ALP assay. The hBMSCs were sprayed at a density of 3.5 × 10⁴ cells/well in a 24-well cell culture plate and then cultured in osteogenic medium. The osteogenic medium was made up of complete α-MEM, 50 μg/mL ascorbic acid (Sigma-Aldrich, St. Louis, MO, USA), 0.1 μM dexamethasone (Sigma-Aldrich), and 10 mM beta-glycerophosphate (Sigma-Aldrich). Individual disks were placed on inserts in the wells. Emdogain at an initial concentration of 30 mg/mL was added to groups 2, 4, 6, and 8, and diluted in osteogenic medium to a final concentration of 100 μg/mL. hBMSCs with experimental disks were incubated for 6 days, with the medium replaced every 48 h. At each observation point, ALP activity was evaluated using Senso-Lyte^®^ p-nitrophenylphosphate (pNPP) alkaline phosphatase assay kit (AnaSpec, Fremont, CA, USA) by the manufacturer’s method. Finally, an absorbance microplate reader was used to measure the optical density at 405 nm. Each group was measured in sextuplicate.

### 2.7. Alizarin Red S (ARS) Staining Assay

The ARS staining assay was used to evaluate the formation of calcium nodules in hBMSCs. Each experimental disk was incubated in osteogenic medium and kept in an incubator at 37 °C and 100% humidity for 7 days, producing a concentration of 5 mg/mL experimental eluate for each tested material. The superficial fluid was purified by 0.20-μm filters (Minisart; Sartorius Stadium Biotech, Göttingen, Germany). hBMSCs were sprayed at a density of 2.0 × 10⁴ cells/well in a 24-well cell culture plate, then incubated for 14 days in the experimental material eluates. Emdogain at an initial concentration of 30 mg/mL was added to groups 2, 4, 6, and 8, and diluted in osteogenic medium to a final concentration of 100 μg/mL. The cells were fixed with a 4% paraformaldehyde and a 2% ARS solution (ScienCell, Carlsbad, CA, USA) for 20 min. The staining was performed for 15 min with 10% cetylpyridinium chromide (Sigma-Aldrich). Finally, an absorbance microplate reader was used to measure the optical density at 560 nm. Each group was measured in sextuplicate.

### 2.8. Statistical Analysis

The SPSS software program (ver. 24.0; IBM Corp., Armonk, NY, USA) was used for statistical analysis. Shapiro–Wilk normality verification was used to evaluate the distribution of the data. After confirming the normality of the data, a repeated-measures analysis of variance (RM ANOVA) was performed for the overall experimental method. If the significance of the group factors was subsequently confirmed, one-way analysis of variance (one-way ANOVA) was implemented and post-validation was performed using the Tukey post hoc test. Differences associated with the application of Emdogain were verified using the paired *t*-test. Differences with values of *p* < 0.05 were considered statistically significant.

## 3. Results

### 3.1. Cell Viability Measurement

**Figure 1:**
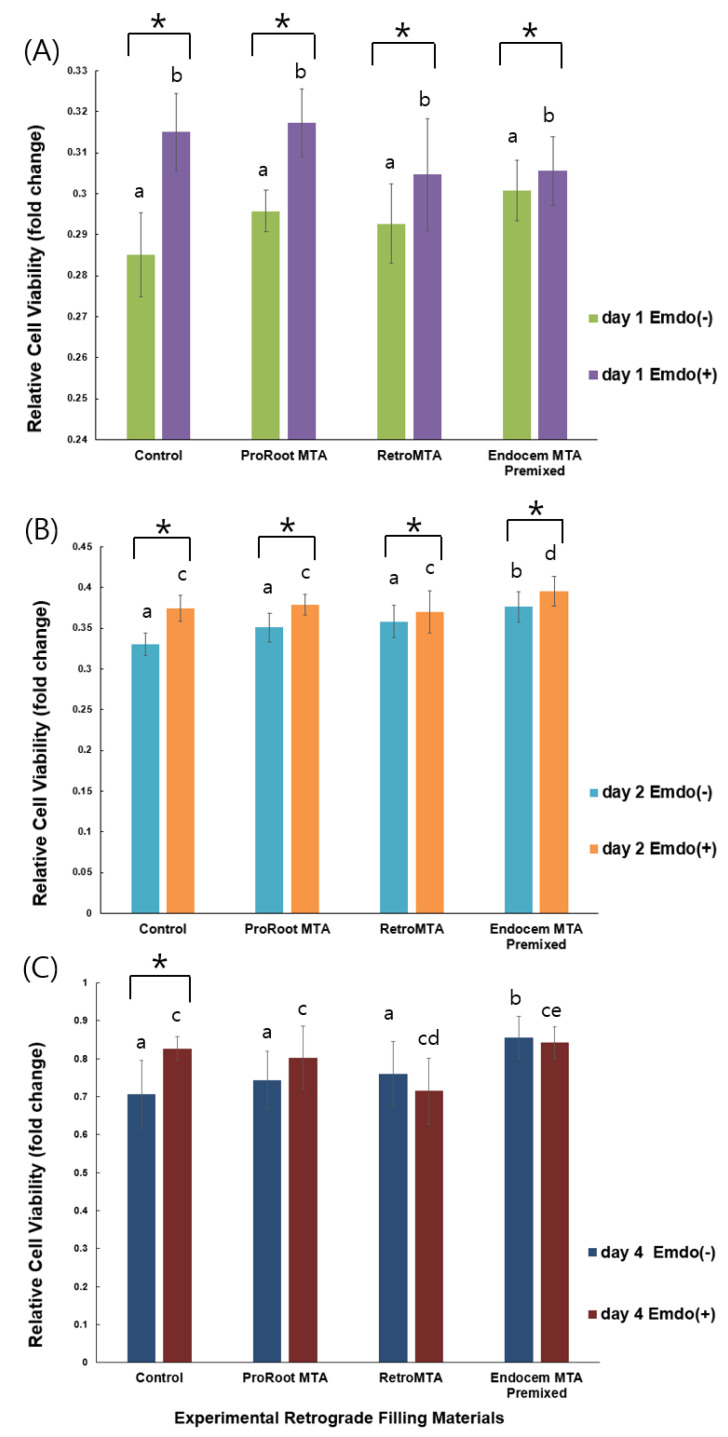
Comparison of cell viability values by material with or without Emdogain application at (**A**) day 1, (**B**) day 2, and (**C**) day 4. Different letters indicate statistically significant differences between the tested materials, and stars indicate that there are statistically significant differences between cells with and without Emdogain application.

There was no significant difference between the experimental groups, with or without Emdogain application, and the control group at day 1 (Figure 1A, *p* > 0.05). Significantly more cell proliferation was observed only in the Endocem MTA Premixed group, with or without Emdogain application, compared with the other groups at day 2 (Figure 1B, *p* < 0.05). At days 1 and 2, all groups treated with Emdogain showed significantly higher cell proliferation compared with those without Emdogain (Figure 1A,B, *p* < 0.05), whereas a significant difference between Emdogain-treated and untreated cells was observed only in the control group at day 4 (Figure 1C, *p* < 0.05).

### 3.2. Cell Migration Assay

**Figure 2:**
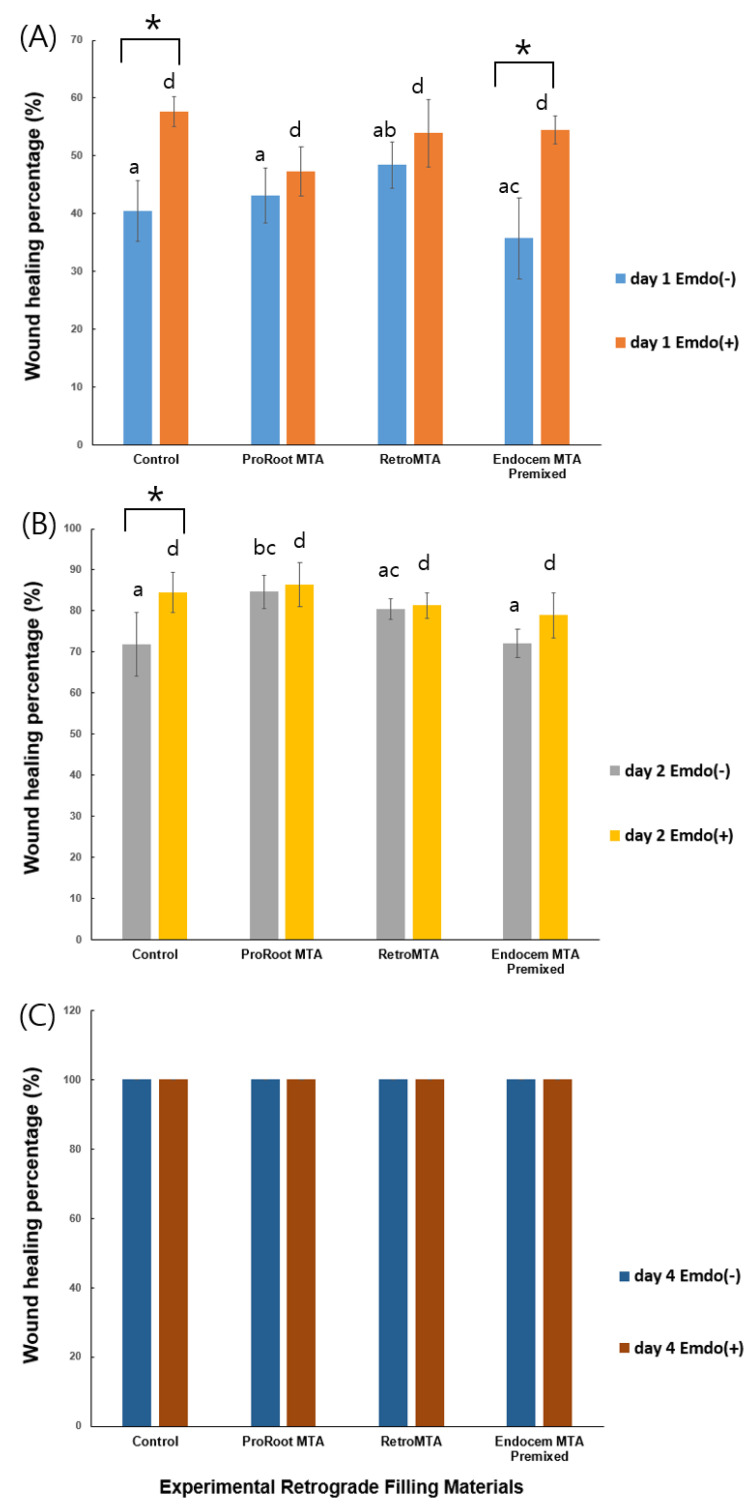
Comparison of cell migration values by material with or without Emdogain at (**A**) day 1, (**B**) day 2, and (**C**) day 4. Different letters indicate statistically significant differences between the tested materials, and stars indicate that there are statistically significant differences between cells with and without Emdogain application.

**Figure 3:**
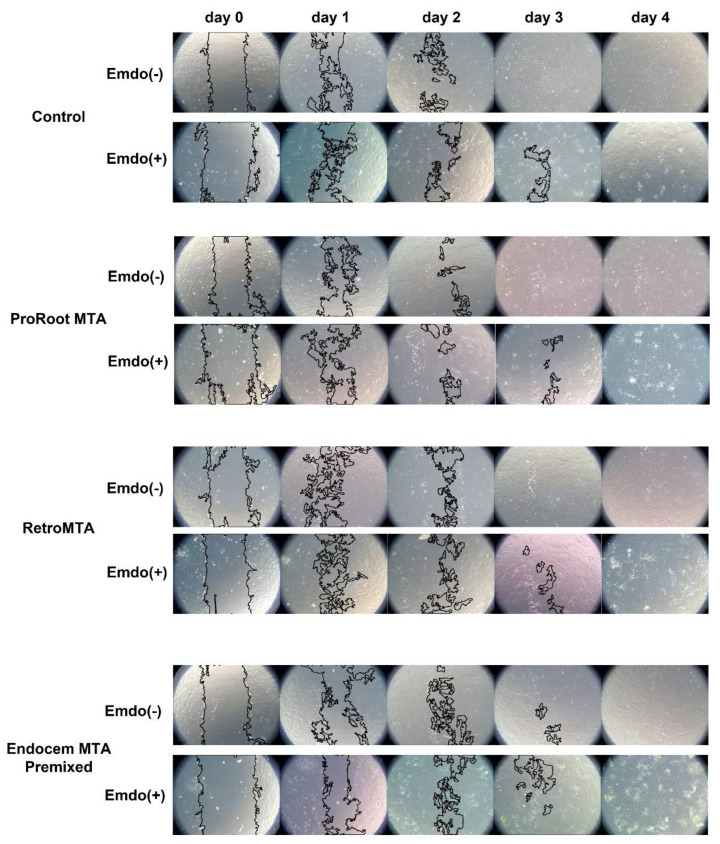
Representative images from the cell migration assay.

Regardless of Emdogain application, wound-healing values increased significantly over time, with no material-specific effects (Figure 2). The control and Endocem MTA Premixed groups with Emdogain presented significantly higher wound healing percentages at day 1 (Figure 2A, *p* < 0.05), as did the control group alone at day 2 (Figure 2B, *p* < 0.05). At day 4, all groups showed almost complete wound healing. Representative images of each group are shown in Figure 3.

### 3.3. Alkaline Phosphatase (ALP) Activity

**Figure 4:**
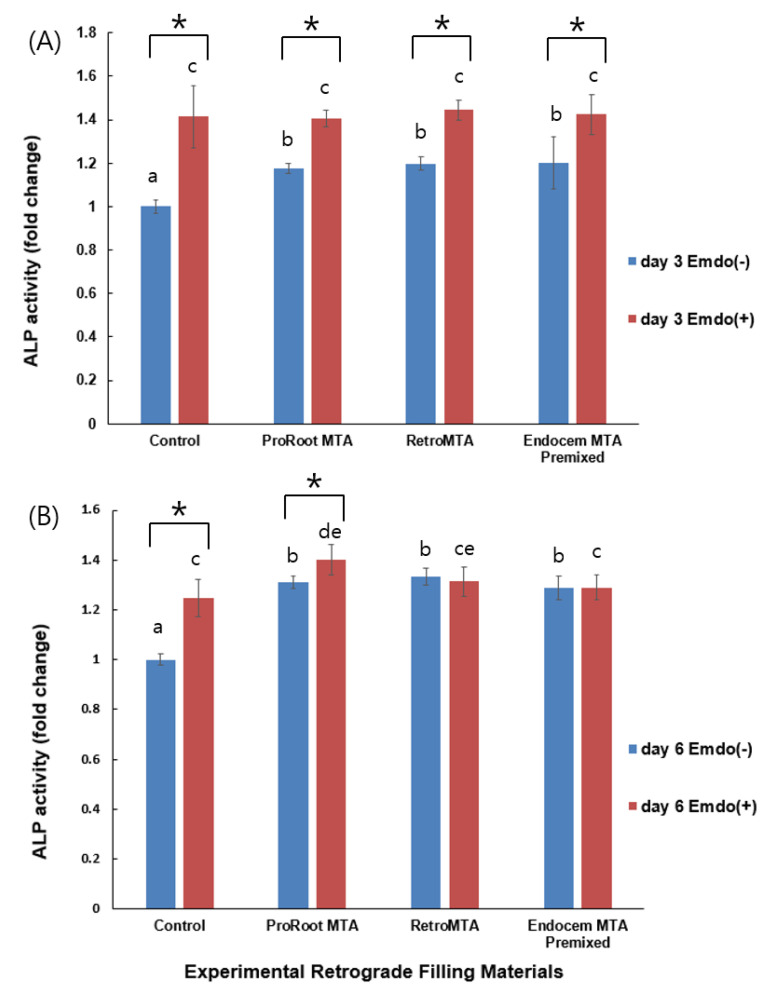
Comparison of ALP activity by tested material with or without Emdogain at (**A**) day 3, and (**B**) day 6. Different letters indicate statistically significant differences between the tested materials, and stars indicate that there are statistically significant differences between cells with and without Emdogain application.

At days 3 and 6, there were no differences between the experimental groups without Emdogain application, and all the experimental groups showed higher ALP activity than that of the control group (Figure 4). At day 3, all groups with Emdogain application showed significantly higher ALP activity than those without Emdogain (Figure 4A, *p* < 0.05), whereas only the control and ProRoot MTA groups did at day 6 (Figure 4B, *p* < 0.05).

### 3.4. Alizarin Red-S (ARS) Staining Assay

**Figure 5:**
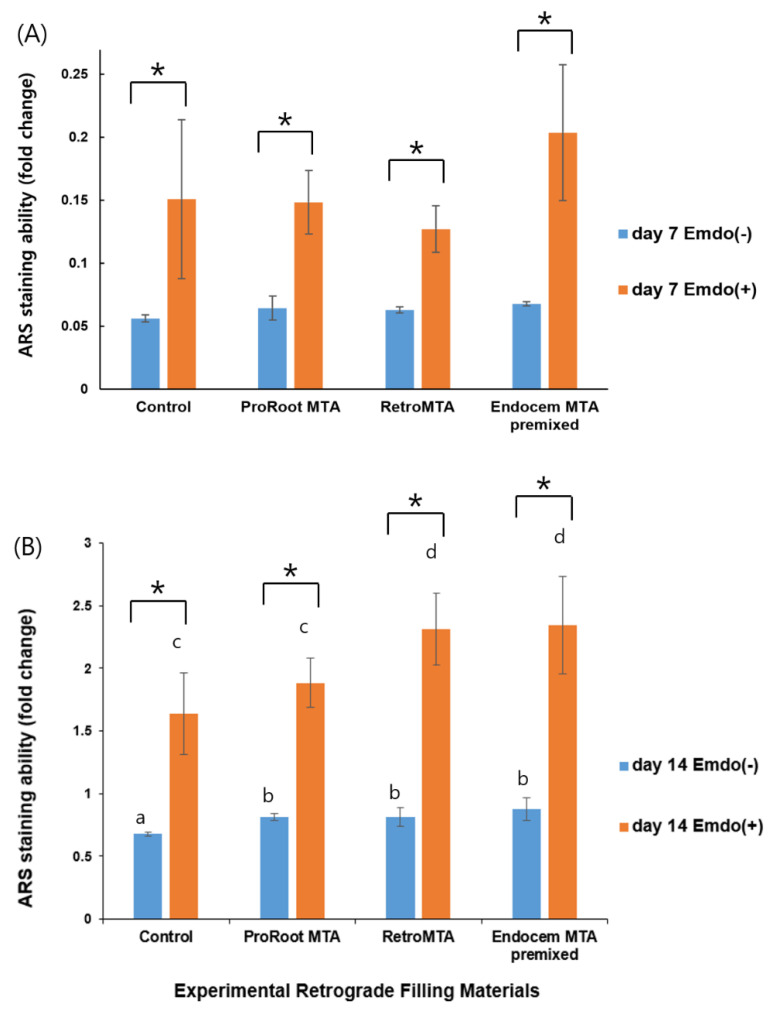
Comparison of ARS staining by material with or without Emdogain at (**A**) day 7, and (**B**) day 14. Different letters indicate statistically significant differences between tested materials, and stars indicate that there are statistically significant differences between cells with and without Emdogain application.

**Figure 6:**
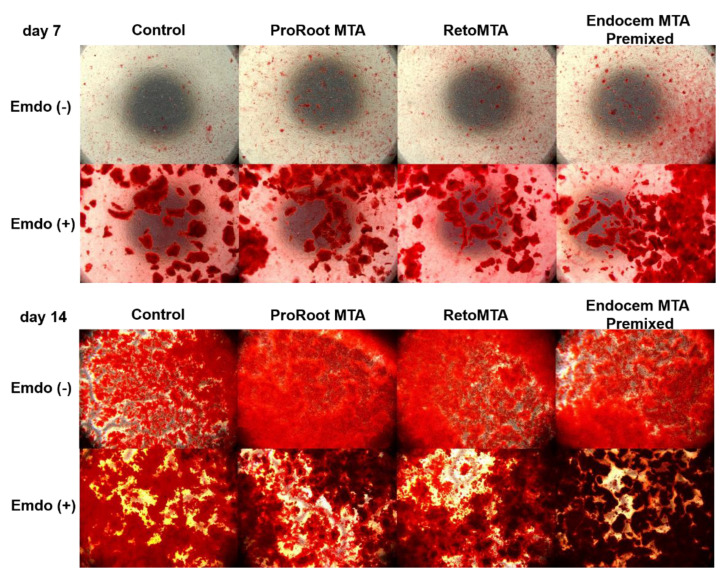
Representative images from the ARS staining assay.

Overall, we observed increased ARS staining at day 14 compared with that at day 7 (Figure 5). There were no material-specific differences at day 7. However, all groups treated with Emdogain presented significantly higher ARS staining than those without Emdogain (Figure 5A, *p* < 0.05). At day 14, especially when Emdogain was applied, the ARS staining of the RetroMTA and Endocem MTA Premixed groups was significantly higher than that of the ProRoot MTA and control groups (Figure 5B, *p* < 0.05). Representative images of each group are shown in Figure 6.

## 4. Discussion

MTA was introduced to facilitate bone regeneration, due to its excellent biocompatibility and antibacterial and sealing ability in apical microsurgery [4]. However, because bleeding in the surgical field may inhibit the hardening of MTA, CSCs have been developed to overcome this problem [15,16,17,18]. In addition, the use of EMD to promote pulp stem cell proliferation and hard tissue regeneration in endodontic dentistry has recently been reported [26,27,28,29,30]. Conventionally, EMD was considered to be a material with great potential to enhance the regeneration of periodontal tissue [22,23,24,25]. In this study, we compared the biocompatibility and osteogenic potential of ProRoot MTA, RetroMTA, and Endocem MTA Premixed combined with Emdogain on hBMSCs.

We assayed CCK-8 analysis and a wound-healing assay to evaluate the biocompatibility of ProRoot MTA, RetroMTA, and Endocem MTA Premixed. Biocompatibility is the first essential factor in cell differentiation and the production of extracellular mineralized materials. The most commonly used cell viability assays are tetrazolium reduction, resazurin reduction, and the protease activity assay, all of which measure the general metabolism or enzymatic activity of viable cells [31]. Methyl thiazol tetrazolium (MTT) chromatic analysis has been widely used as a standard technique for assessing the cytotoxicity of biomaterials [32,33,34]. However, the MTT assay underestimates cell damage and can detect cell death only at the later stage of apoptosis when cell metabolism is significantly reduced [35]. To evaluate the cytotoxic effect of various CSCs on hBMSC, we used the CCK-8 method, which is known for its superior sensitivity and low cytotoxicity compared with the MTT assay and is also convenient for measuring the same sample repeatedly [36]. Cell migration to scratch sites is an important indicator of a material’s biocompatibility [37].

In this study, biological responses showed that there was no significant difference between the ProRoot MTA, RetroMTA, Endocem MTA premixed, and control groups without Emdogain application. In other words, neither MTA nor CSCs had a detrimental effect on cell survival, but Endocem MTA Premixed provided significantly higher cell viability than the other treatments and the control at days 2 and 4 (Figure 1B,C, *p* < 0.05). When Emdogain was added, significant differences were observed in the early stages of the experiment (days 1 and 2), but the effect was not meaningful later. Thus, ProRoot MTA and RetroMTA are similarly biocompatible with hBMSCs, and cells cultured with Endocem MTA Premixed proliferated more than those in the other groups at days 2 and 4. The addition of Emdogain improved the biological responses in comparison with those achieved without Emdogain in all the experimental groups, but the differences decreased over time.

Previous studies have found that the cell viability achieved using RetroMTA is similar to or higher than that of a control group [19,20]. Other studies have also reported superior or similar biocompatibility of human dental pulp stem cells (hDPSCs) when treated with ProRoot MTA [38,39], consistent with our results. In a previous study that compared the dental pulp stem cell (DPSC) biocompatibilities of ProRoot MTA and Emdogain using an MTT assay [26], Emdogain showed the least cytotoxicity to DPSC, with 77% cell viability, followed by ProRoot MTA with 53%. Moreover, Wang et al., [30] found that EMD enhances the mineralization activity of DPSC by upregulating odontoblast or osteoblast induction markers. The results of these studies are also in accord with ours; therefore, EMD could be an alternative or adjuvant material to MTA. On the other hand, other studies have reported opposite results. In the study of Youssef et al., ProRoot MTA gave significantly lower cell viability compared with Emdogain [26]. In addition, a prior study by Bortoluzzi et al., [40] showed that eluates of MTA Angelus (Angelus Dental Solutions, Londrina, PR, Brazil) and Biodentine (Septodont, Saint Maur-des-Fosses, France) affect cell viability. The reason for this was not clear, but early release of calcium ions, ionic activity, toxic components, or pH changes might affect cells [41,42].

Osteogenic potential plays an important role in bone regeneration around teeth. In this study, we used ALP activity and an ARS staining assay to evaluate osteogenic potential. The ARS staining assay revealed significantly higher calcium nodule deposition at day 14 in the groups with MTA or CSCs than in the control group, and the effects of adding Emdogain were also greater. Regarding ALP activity, the groups with MTA or CSCs at days 3 and 6 had higher ALP levels than the control group. This demonstrates that MTA and CSCs increased the cells’ osteogenic potential, and that the additional application of Emdogain would result in a higher degree of mineralization.

Santiago et al., reported a significant increase in ALP activity in a ProRoot MTA group compared with their control group. The bone sialoprotein (*BNSP*) gene, a marker of osteogenesis, was significantly overexpressed in the ProRoot group [43]. A study using hDPSCs exposed to ProRoot MTA indicated that high pH and the release of calcium ions can influence the differential expression of the osteogenesis markers BGLAP and BMP-2 [44]. Similarly, a previous study showed that the contact of ProRoot MTA and water produces calcium hydroxide, which leads to the release of calcium ions [20]. These studies all support our current finding that the use of ProRoot MTA with or without Emdogain resulted in higher ALP activity at days 3 and 6, and higher ARS staining at day 14. In the ALP analysis, the values in all groups treated with Emdogain were significantly higher only at day 3, whereas they were only higher in the control and ProRoot MTA groups at day 6. This means that in the groups treated with CSCs, such as RetroMTA and Endocem MTA Premixed, Emdogain can be thought to have an early effect on bone tissue formation at day 3. As the influence of Emdogain declined over time, CSC alone was sufficient to support osteogenic potential at day 6.

When Emdogain was applied, calcium nodule deposition levels were significantly higher in all experimental groups and the control group, especially in the RetroMTA and Endocem MTA Premixed groups at day 14 (Figure 5B). Unlike the ALP activity results, the ARS results suggest that Emdogain continuously strengthened the bone tissue mineralization process during the 14 days of the experiment. In particular, components of the CSCs used in this study seemed to have more influence on hard tissue formation than ProRoot MTA, by continuously releasing calcium ions. The continuous release of calcium ions and silica components can affect bone formation [45,46]. Silica has been claimed to be an essential component of the CSC mechanism of action, inhibiting the formation of osteoclast and bone resorption [47]. For this reason, the ability to form calcium nodules continued to be higher in the CSC groups compared with the ProRoot MTA and control groups, until day 14. In this study, Emdogain also played an auxiliary role in the hBMSC mineralization process. Ishizaki et al., reported that odontoblasts and pulp cells are stimulated directly by Emdogain to produce collagen substrates for calcification [48]. Moreover, Guven et al., proved that EMD increases ALP activity and DSPP gene expression in comparison with ProRoot MTA [49]. The authors suggested that EMD increases hard tissue regeneration and can be used in pulp-capping procedures [49]. Other studies have also shown that Emdogain promotes bone and cementum production [50,51,52,53]. Kawase et al. suggested that amelogenin peptide or transforming growth factor-β1 present in Emdogain is involved in cell signaling to stimulate substrate formation and mineralization [51,52].

The components from the material spread to the medium at different speeds, and this may indicate concentrations of the ingredients that have different effects. This may explain why the results of this in vitro study demonstrated differences between materials in their overall effectiveness on hBMSC. Therefore, in vivo research will be the next step to more accurately determine the ultimate impact of these substances and cells’ reactions to them. Defining the clinical relevance of the findings is expected to require further research. However, despite these limitations, the results of this study justify using the various CSCs developed to overcome the disadvantages of MTA as retrograde filling materials during apical microsurgery. The additional application of Emdogain is expected to help with early cell proliferation and osteogenic potential, and therefore will increase the regeneration at the site of apical bone destruction.

## Figures and Tables

**Table 1:** Materials used in this study, their manufacturers, and chemical compositions.

Material	Manufacturer	Composition	Batch Number
ProRoot MTA	Dentsply Tulsa Dental Specialties, Tulsa, OK, USA	Portland cement (tricalcium silicate, dicalcium silicate, and tricalcium aluminate) 75% Calcium sulfate dihydrate (gypsum) 5% Bismuth oxide 20%	0000186484
RetroMTA	BioMTA, Seoul, Korea	Calcium carbonate 60–80% Silicon dioxide 5–15% Aluminum oxide 5–10% Calcium zirconia complex 20–30%	RM1810D14
Endocem MTA Premixed	Maruchi, Wonju, Korea	Natural pure cement Bismuth trioxide	C2304160716
Emdogain Gel	Straumann, Basel, Switzerland	Amelogenin 90% The remainder is proline-rich nonamelogenin, tuftelins, tuft proteins, ameloblastin, and amelins	ISO 15223-1

## Data Availability

The datasets used and/or analyzed during the current study are available from the corresponding author on reasonable request.
